# The Malaysian Burden of Disease Study: Protocol for Mortality, Morbidity, and Risk Factor Evaluation

**DOI:** 10.2196/71583

**Published:** 2025-11-14

**Authors:** Wan-Fei Khaw, Nazirah Alias, Sin Wan Tham, Kim Sui Wan, Nur Hamizah Nasaruddin, Nur Diyana Rosnan, Shubash Shander Ganapathy, Mohd Azahadi Omar

**Affiliations:** 1 Institute for Public Health National Institutes of Health Ministry of Health Malaysia Shah Alam, Selangor Malaysia; 2 Institute for Medical Research National Institutes of Health Ministry of Health Malaysia Shah Alam, Selangor Malaysia

**Keywords:** Malaysia, disease burden, disability-adjusted life years, risk factors, population health

## Abstract

**Background:**

Knowledge of the burden of disease at the national level is crucial for understanding regional health patterns and epidemiological trends. Regular updates and methodological enhancements are essential to produce accurate estimates that effectively represent the current health landscape, thereby assisting policymakers in resource allocation and strategic planning.

**Objective:**

The objective of this study protocol is to revise the list of diseases and injuries; estimate years of life lost (YLLs), years lived with disability (YLDs), and disability-adjusted life years (DALYs) in phase 1; and identify the list of risk factors and determine risk-attributable burdens in phase 2.

**Methods:**

The Malaysian Burden of Disease (MBOD) Study adopts the Global Burden of Disease Study approach, using various metrics to quantify health loss related to specific diseases, injuries, and risk factors. These metrics include deaths, YLLs, YLDs, and DALYs, which are calculated in terms of counts, age-specific rates, and all-age rates. In addition, this MBOD Study calculates risk-attributable deaths, YLLs, YLDs, and DALYs for all relevant risk factors. This study also conducts focus group discussions to develop the lists of diseases and injuries as well as risk factors.

**Results:**

By December 2024, we had successfully completed the focus group discussions, which led to the development of an updated and comprehensive list of diseases and injuries. Furthermore, mortality data collection is complete, and the calculation of YLLs is currently in progress. The YLD calculation is expected to conclude by the end of 2025. While phase 1 is underway, phase 2, which addresses risk factor estimation, is scheduled for completion in mid-2026.

**Conclusions:**

This MBOD Study offers a comprehensive and current framework for assessing the burden of disease in Malaysia, with continuous improvement initiatives to enhance the list of diseases and injuries and refine the methodology for more accurate estimation and risk factor analysis.

**International Registered Report Identifier (IRRID):**

DERR1-10.2196/71583

## Introduction

### Background

The Global Burden of Disease (GBD) Study uses published and publicly accessible data to provide an extensive evaluation of incidence, prevalence, and mortality for a wide range of diseases [1,2]. Disability-adjusted life years (DALYs) quantify healthy life lost due to diseases and injuries, combining mortality, measured as years of life lost (YLLs), and morbidity, measured as years lived with disability (YLDs) [3]. The GBD Study applies a standardized framework that enables national-level quantification and assessment of the burden of disease across more than 200 countries [3]. However, the GBD Study relies primarily on global modeling methods, with limited use of localized data, which may impact the granularity and accuracy of its findings for individual countries.

The Malaysian Burden of Disease (MBOD) project was initiated by the Institute for Public Health, Ministry of Health Malaysia, to close this gap. The first estimates were produced for the year 2000, and the most recent completed MBOD Study covers the year 2019. The project’s goal is to evaluate the burden of disease at the national level while providing a regional perspective on health trends and epidemiological shifts [4-6].

Among the most common causes of disease burden among Malaysians, noncommunicable diseases (NCDs) continue to be a major public health concern [7]. Since 2021, COVID-19 has also emerged as a major contributor to the national burden of disease [3], which highlights the need for up-to-date data. However, no recent study has been conducted to assess the impacts of COVID-19 and NCDs burden trends.

Knowledge of the burden of disease is important for developing relevant and effective public health policies, particularly in a pandemic-impacted health system. Current and detailed information on disease burden is critical for identifying emerging health concerns and highlighting diseases that cause the most fatalities and preventable deaths. The MBOD project has made significant progress in its methodologies and scope over the past 2 decades [8-10]. The availability and granularity of data have substantially increased, and the list of diseases and injuries assessed has expanded, providing more detailed insights [5]. Long-standing gaps in the accuracy of mortality statistics have been successfully addressed by methodological improvements such as the use of verbal autopsy data and the redistribution of ill-defined causes of death [5,11]. Moreover, the MBOD Study continues to generate findings that are highly relevant for shaping public health policies and decision-making by integrating localized data and highlighting risk-attributable burden. Importantly, this protocol represents a significant advancement in Malaysia, as it is the first framework to include calculations of risk-attributable burden in MBOD studies.

The comparative risk assessment tool, developed by the GBD project, is a useful way of quantifying the burden of disease attributable to various risk factors [1,12]. By using standardized and internationally comparable measurements, the MBOD project’s inclusion of components on risk factors, mortality, and morbidity fills important knowledge gaps. This comprehensive approach not only strengthens the MBOD research protocol but also allows for more accurate estimations of Malaysia’s burden of disease. The MBOD Study’s findings on risk factors provide insights into the modifiable risk factors contributing to health loss, helping to identify the determinants of mortality and morbidity and providing a road map for targeted public health interventions and resource allocation.

### Objectives

This study aims to provide a comprehensive and up-to-date assessment of the burden of disease in Malaysia for the year 2023. The primary objective will be addressed in 2 phases: phase 1 focuses on revising the list of diseases and injuries and estimating YLLs, YLDs, and DALYs, while phase 2 aims to identify risk factors and determine their attributable burdens.

## Methods

### Overview

**Figure 1:**
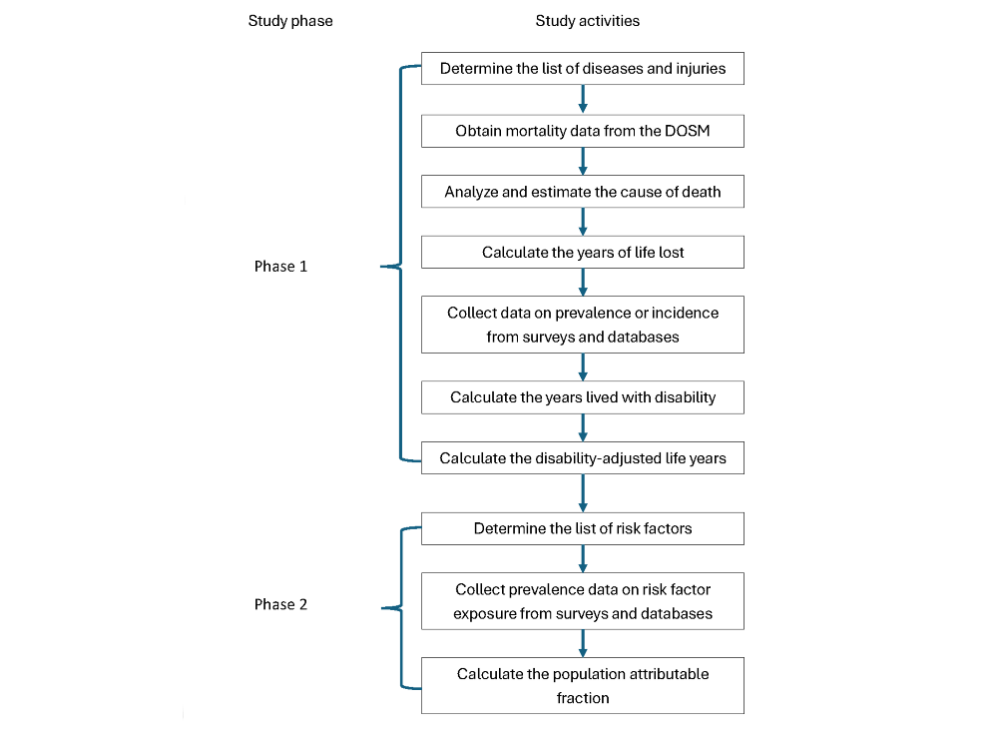
Flowchart of the study.

The study methodology involves selecting diseases, injuries, and risk factors, followed by estimating deaths, YLLs, YLDs, and DALYs from diseases and injuries as well as risk-attributable deaths, YLLs, YLDs, and DALYs. All metrics are calculated by age and sex across 18 age groups (from birth to 80 years or older) and are disaggregated by male individuals, female individuals, and both sexes combined. These metrics are calculated in counts, age-specific rates, and all-age rates focusing on data for the year 2023. An overview of the study workflow is presented in Figure 1. Phase 1 focuses on estimating mortality and morbidity. This involves identifying the relevant list of diseases and injuries as well as gathering and analyzing data. Phase 2 will focus on risk factor analysis. This includes identifying the list of risk factors, collecting exposure data, and performing data analysis to determine risk-attributable burden.

### Determination of Reference Year (2023)

The year 2023 was chosen because updated prevalence data from the National Health and Morbidity Survey conducted that year [13] are available and essential for our calculations. In addition, following the stabilization of data after several years of the COVID-19 pandemic, 2023 serves as a reliable baseline for forecasting future trends. Furthermore, this study integrates newly available data sources and applies refined methodological approaches for mortality estimation.

### Determination of the Lists of Diseases, Injuries, and Risk Factors to be Included in the Study

#### Overview

The lists of diseases, injuries, and risk factors for the MBOD Study are guided by the GBD Study framework. The GBD disease and injury list is organized into 4 hierarchical levels: level 1 includes 3 broad categories (communicable, maternal, neonatal, and nutritional diseases; NCDs; and injuries); level 2 further categorizes these into 22 categories; and level 3 and level 4 comprise 174 and 301 specific causes, respectively. Across all levels, there are a total of 369 diseases and injuries [2]. The GBD risk factor list is also structured into 4 levels: level 1 includes behavioral, environmental and occupational, and metabolic categories, followed by level 2 with 20 individual risks or clusters, level 3 with 52 risk factors or clusters, and level 4 with 69 specific risk factors. Across all levels, there are a total of 87 risk factors [12].

#### Focus Group Discussions

To adapt the GBD lists to the Malaysian context, focus group discussions are conducted to evaluate the relevance of diseases, injuries, and risk factors, along with the availability of associated data. Focus group discussions have been chosen as the data collection method because they allow for interactivity between participants in focused discussions guided by researchers [14]. This approach can generate critical insights for clinicians, experts, and policymakers regarding the importance of diseases, injuries, and risk factors relevant to the local context.

Purposeful sampling is used to select individuals based on their capacity to provide relevant information in response to the research questions. Participants are selected based on their expertise and experience in related disciplines. One expert is selected from each disease group or risk factor category to ensure representation and comprehensive input across all relevant areas. These experts, including specialists, consultants, and subject matter experts, are invited to contribute their perspectives. They are contacted by letter and follow-up telephone calls, with a clear explanation of the study’s objectives provided to potential participants. No further inclusion or exclusion criteria are applied.

To develop a comprehensive and contextually relevant list of diseases and injuries for the MBOD Study, 10 focus group discussions were carried out to discuss the relevance of diseases and the availability of data for each condition. Each group consisted of 7 to 12 experts [14], including disease burden experts, disease experts, and researchers. These sessions were moderated by an experienced researcher who facilitated discussions aimed at gathering opinions and recommendations and ultimately achieving consensus on the inclusion and prioritization of diseases and injuries for the MBOD Study. During these focus group discussions, participants were asked to evaluate each disease or injury based on several criteria, including the probable magnitude of the disease or injury (≥0.01%) as a cause of death, the level of health services associated with the disease or injury, the public health importance of the disease or injury in the local context, the availability of morbidity data, and the relevance of that specific disease or injury to policy in Malaysia. In addition, the availability of local data sources was reviewed to ensure that the disease or injury could be reliably quantified and used in the calculations.

For the selection of the risk factor list, 3 additional focus group discussions will be organized to discuss the relevance of, and data availability for, each risk factor. Participants will be grouped by their field of expertise and according to level 1 risk factor categories. Each focus group comprises 7 to 12 participants. The sessions are moderated by experienced researchers who guide the discussions. The discussions aim to gather opinions and recommendations and ultimately reach an agreement on developing the risk factor list for the MBOD Study. The inclusion of risk factors is prioritized based on nationwide relevance, clarity of identification without excessive specificity, availability of evidence linking them to disease causation, accessibility of information on their distribution across the population, and their modifiability. This structured methodology ensures that the final lists of diseases, injuries, and risk factors are both scientifically rigorous and contextually relevant for the MBOD Study.

### Calculation of YLLs

Mortality data from January to December 2023 will be included in this study. Data on deaths, including sex, age, and cause of death, will be obtained from the Department of Statistics Malaysia (DOSM). All data provided by the DOSM will have personal identifiers removed to comply with Malaysia’s Personal Data Protection Act.

### Improvements to Cause-of-Death Data Estimation in the MBOD Study 2023

#### Improvements in Redistribution of Nonmedically Certified Deaths

In the MBOD Study 2023, we will make improvements to the redistribution process for nonmedically certified deaths to enhance the accuracy of mortality data. It is important to note that nonmedically certified deaths do not capture all diseases and injuries; only certain causes are included. To address this limitation, we will apply proportions derived from medically certified deaths to redistribute causes of nonmedically certified death cases that lack specificity. In addition, we will apply cause-specific mortality fraction obtained from verbal autopsy to ensure more accurate representation of the causes of death. Subsequently, age group distribution and the envelope adjustment technique will be applied to the nonmedically certified death data. The purpose of these procedures is to ensure that the total number of deaths is consistent across specific sex and age groups.

#### Expansion of Disease and Injury List and Inclusion of COVID-19 Estimates

In the MBOD Study 2023, the list of diseases and injuries included in the estimating framework will be expanded and revised. Adding conditions and diseases not previously included allows for a more complete evaluation of mortality and morbidity. The selection of diseases and injuries is based on international guidelines and local health goals. In addition, COVID-19 will be included in the estimating procedure because of its significant impact on population health, highlighting the pandemic’s effect on recent death trends and ensuring that this study is current and relevant. Adding COVID-19–specific estimates and expanding the disease and injury list enhances the accuracy and comprehensiveness of Malaysia’s burden of disease estimates.

### Steps for Determining Causes of Death

Mortality data will be classified into 2 categories: medically certified deaths and nonmedically certified deaths. Medically certified deaths occur in medical facilities, and the cause of death is certified by the attending physician. Nonmedically certified deaths occur outside of medical facilities and are reported by the family of the deceased to the local police, who provide nonexpert opinions on the cause of death.

To enhance the quality of mortality data, we will use cause-specific mortality fraction for nonmedically certified deaths, derived from verbal autopsy, to reduce the number of ill-defined and nonspecific causes of death [9]. When there is insufficient information to determine the exact cause of death, we will categorize the causes of death under “garbage codes.” It should be noted, however, that garbage codes compromise the usefulness of cause-of-death information from a policy perspective. To address this, MBOD experts will analyze the garbage codes listed by the World Health Organization and GBD studies [5]. Subsequently, ill-defined causes of death will undergo reclassification, and redistribution methods will be used to refine the data. The analysis will calculate the number of deaths attributed to each disease, disaggregated by age and sex groups.

### Sensitivity and Uncertainty Analyses

#### Overview

To verify the stability and robustness of the results, a sensitivity analysis will be conducted by comparing the distribution of mortality data with and without nonmedically certified deaths. This analysis aims to ensure that the redistribution process produces cause-of-death patterns consistent with those observed in medically certified deaths across the major disease categories.

The same technique used in the GBD Study will be applied in this study to quantify uncertainty. Monte Carlo simulation will be performed using R software (R Foundation for Statistical Computing). Final estimates will be generated as the average estimate of 1000 samples. The 95% uncertainty interval will be derived based on the 2.5th and 97.5th percentiles of the bit-sorted distribution of 1000 samples.

#### Steps for Calculating YLLs

Relevant R packages will be used to calculate deaths and YLLs. The R packages that will be used in the MBOD Study 2023 include *dplyr* and *tidyr* for data manipulation, *readxl* and *openxlsx* to read the data obtained from the DOSM, and *ggplot2* for generating data visualizations. The calculation of premature mortality in Malaysia for 2023 will follow the methodologies developed by Murray and Lopez [15] for the GBD Study to compute YLLs [2]. YLLs will be determined by multiplying the number of deaths attributed to a specific disease by the remaining life expectancy for the corresponding age and sex group. Life expectancy data will be obtained from DOSM life tables.

### Calculation of YLDs

#### Sources of Morbidity Data

Prevalence estimates for each disease and injury will be identified from all potential data sources (eg, national surveys, surveillance reports, census records, monitoring programs, disease registries, and hospitalization records) and assessed for comparability, relevance, representativeness, currency, accuracy, creditability, and accessibility or timeliness. A data quality assessment matrix will be used to evaluate all morbidity data sources. This matrix is modeled on the Australian Burden of Disease Study, which prioritizes the use of the highest-quality data available [16]. In instances where local data are lacking, data from meta-analyses, systematic reviews, or the Global Health Data Exchange (created and hosted by the Institute for Health Metrics and Evaluation) using statistical modeling will be used, with DisMod II (version 1.04) applied to re-estimate local relevance.

#### Steps for Calculating YLDs

YLDs represent the impact of nonfatal diseases and injuries. Prevalence estimates for each disease and injury, which include a breakdown of the severity proportion and percentage contributing to the sequelae, will be calculated. The YLD calculation will be based on this prevalence and a set of disability weights for each condition obtained from the GBD 2019 Supplement [2]. Data sources for YLD calculations will include locally available surveys and hospital discharge records. When relevant data sources are not available, the Global Health Data Exchange and more specifically the GBD Study results will be used to obtain the incidence or prevalence statistics. These data will then be processed using DisMod II to generate prevalence estimates by taking into account factors such as incidence, prevalence, remission, case fatality, duration, mortality, and relative risk (RR) mortality, based on a limited amount of local data [17].

Microsoft Excel and relevant R packages will be used to calculate YLDs. In addition to the previously mentioned R packages, *lubridate* will be used to handle time calculations for YLD data. The YLD will be determined by multiplying the prevalence of each disease sequela by its disability weight [15], stratified by age and sex. The total YLD for each disease and injury will be derived by summing the YLDs from all sequelae associated with that disease or injury.

### Calculation of DALYs

DALYs provide a comprehensive measure of the burden of disease and injury by combining YLLs and YLDs. For each disease and injury, the DALY will be calculated as the sum of YLL and YLD. The overall burden of disease will then be determined by aggregating DALYs across all diseases. Calculations will be performed using relevant R packages.

### Calculation of Risk-Attributable Burden

#### Sources of Risk Factor Prevalence Data

Prevalence estimates for each risk factor exposure will be identified from all potential data sources (eg, national surveys, surveillance reports, census records, and monitoring programs) and assessed for comparability, relevance, representativeness, currency, accuracy, creditability, and accessibility or timeliness. A data quality assessment matrix will be used to evaluate all sources. This matrix is modeled on the Australian Burden of Disease Study, which prioritizes the use of the highest-quality data available [16].

#### Steps for Calculating Risk-Attributable Burden

In this study, linked diseases are conditions from the disease list that are associated with known risk factors. We will adopt the relevant linked diseases from the GBD Study 2019 framework [12] and review the linked diseases corresponding to selected risk factors to ensure local relevance. Malaysia-specific exposure distributions and contextually relevant RR estimates from local cohort studies, surveys, or administrative datasets will be prioritized. In the absence of local RR data, we will rely on global estimates while recognizing the limitations and potential variability in risk-outcome relationships.

GBD-linked risk-outcome pairs from the GBD Study will be adapted as the foundational framework. The list of risk factors will be refined to reflect Malaysia-specific exposures and epidemiological patterns; for example, dietary risk will be adapted to include high palm oil consumption [18]. To ensure local relevance and modification, the selection of risk-attributable burdens will be discussed in focus group discussions with expert consultants and stakeholders to link disease risk and outcome.

To estimate the attributable burden for each risk-outcome pair, the GBD methodology requires four key inputs: (1) the burden estimate for the outcome (eg, number of deaths, YLL, and DALY), (2) spatially and temporally resolved exposure estimates for the risk factor, (3) the counterfactual or theoretical minimum risk exposure level (TMREL), and (4) the RR or exposure-response relationship for the outcome relative to the TMREL [12]. We will calculate the population attributable fraction (PAF), which represents the proportion of the outcome that, for a given population and year, would be eliminated if the risk factor were reduced to the TMREL. The risk-attributable burden will then be estimated for each risk-outcome pair by age group and sex. Relevant R packages will be used to calculate the PAF.

Uncertainty will be addressed using interval estimates. Specifically, 95% uncertainty intervals for prevalence data and RR values will be generated through Monte Carlo simulation.

### Ethical Considerations

This study was approved by the Medical Research and Ethics Committee, Ministry of Health Malaysia (NMRR ID-24-01855-INU). It involves secondary data analysis, with raw mortality and morbidity data obtained with written permission from the director of the DOSM, the Ministry of Health Malaysia, and through various surveys. All researchers adhere to the principles of the Declaration of Helsinki and the Malaysian Good Clinical Practice Guidelines, where applicable. This study complies with Malaysia’s Personal Data Protection Act, with all data obtained in deidentified form.

## Results

The MBOD Study 2023 consists of 2 phases: phase 1 focuses on mortality and morbidity estimation, and phase 2 focuses on risk factor estimation. As of December 2024, phase 1 is underway, with phase 2 scheduled for completion in mid-2026. Focus group discussions with clinicians (19/37, 51%) and policymakers (18/37, 49%) have been completed, leading to the development of an updated list of diseases and injuries. Mortality data collection has also been completed, and the calculation of YLLs is currently in progress. Phase 1, including YLD calculation, is expected to be completed by the end of 2025, while phase 2 (risk factor estimation) is scheduled to begin thereafter and conclude by mid-2026.

## Discussion

### Summary

This is the sixth MBOD Study since the first one for the year 2000 and marks a significant milestone as the first to provide a comprehensive analysis of both disease burden and risk factors. It addresses a major knowledge gap by using standardized and internationally comparable metrics to analyze the burden of disease, encompassing premature death, morbidity and disability, and risk factors, that contributes to poor health in the Malaysian population.

The MBOD Study has provided regular updates on population health in Malaysia since its first publication. With each subsequent cycle, both the methodology and data sources used to estimate the burden of disease have been substantially updated. For the year 2000, mortality and morbidity estimates were reported for 103 diseases and injuries. By the most recent cycle (2015-2017), these estimates had expanded to include 113 diseases and injuries [6]. Another MBOD project is currently in progress, using new disease and injury list for the year 2019, reflecting a more comprehensive and locally relevant representation of health conditions in Malaysia.

Preliminary findings from our study seem consistent with trends observed in prior MBOD cycles, where NCDs have been the dominant contributors to DALYs since the early 2000s. Early estimates suggest that the increasing burden of cardiovascular diseases, diabetes, and cancers is likely to remain the primary driver of the overall disease burden. These patterns align with global trends reported by the GBD Study [3], in which NCDs dominate the health burden in middle-income countries, including Indonesia [19]. A similar trend is seen across other Association of Southeast Asian Nations (ASEAN) countries, where cardiovascular disease is the fastest growing NCD [20]. Among ASEAN countries, Malaysia has the highest age-standardized prevalence rate of cardiovascular diseases, followed by Indonesia, while Singapore has the lowest rate. However, Malaysia’s disease burden pattern also reflects its unique epidemiological transition: although both communicable diseases and injuries are declining, they remain significant contributors to the overall burden [6]. Ischemic heart disease, stroke, and diabetes remain the leading cause of DALYs, while lower respiratory infection has risen in rank compared to the 2015-2017 MBOD estimates, indicating a worsening local trend compared to the global situation [21]. Data from the National Health and Morbidity Survey further reinforce these findings, showing increasing prevalence of obesity, hypertension, and diabetes in Malaysia [13]. These patterns strongly correlate with our preliminary findings on the rising burden of NCDs and highlight the urgency of national action.

Although other countries have reported on the disease burden attributable to various risk factors [22], no study has assessed an extensive list of risk factors applicable to Malaysia or explored the full extent of disease burden attributable to these factors. Only a few studies in Malaysia have quantified the national disease burden attributable to specific risk factors within an integrated framework; for instance, tobacco smoking and excess weight are established risk factors for cancer [23], while overweight and obesity contribute substantially to the overall disease burden [24]. Our study addresses this gap by incorporating a comprehensive set of risk factors and quantifying their associated health burden, thereby providing a more complete picture of the burden of disease in Malaysia.

In addition, the inclusion of COVID-19 and its attributable burden represents a novel contribution, distinguishing this study from previous MBOD reports. Although mortality estimates for COVID-19 have been reported by several sources [25], no previous publications have specifically quantified its morbidity estimates for Malaysia. While the GBD Study 2021 provides global estimates of the COVID-19 burden [3], local DALY estimates due to COVID-19 have not been previously published in Malaysia. This study, for the first time, expands the list of diseases and injuries, incorporates COVID-19, and provides national-level estimates of disease burden attributable to risk factors. By integrating COVID-19 into the analysis, this study offers a more comprehensive understanding of Malaysia’s health priorities in the postpandemic context and underscores the need for resilient, data-driven public health strategies moving forward.

This study demonstrates several strengths that substantially enhance its quality and relevance. The expanded disease list provides a more complete assessment of health conditions, accurately reflecting Malaysia-specific health challenges such as tropical diseases [26]. The study also enhances mortality estimation, particularly in refining data distribution for nonmedically certified deaths. In addition, the study optimizes local data quality through expert consultations and the use of national surveys, administrative data, and disease registry data, ensuring the use of the highest-quality, most representative Malaysian data for estimating the burden of disease and risk factors.

### Limitations

This study has several limitations that should be considered for balanced interpretation of the findings. First, regarding mortality data, only two-thirds of the deaths were medically certified, necessitating redistribution methods for nonmedically certified deaths and deaths coded under garbage codes. Although these methods are standardized within the burden of disease framework, they introduce a degree of uncertainty in cause-of-death estimation. Continued efforts to reduce the proportion of garbage codes and increase the use of verbal autopsy are essential to improve data quality and the accuracy of cause-of-death estimation.

Second, the estimation of YLDs relies heavily on data from national surveys, hospital records, and disease registries, which are often incomplete or outdated in the Malaysian context. As a result, some disease prevalence estimates depend on data extrapolated from the literature or small-scale studies rather than directly observed data. This may limit the accuracy and representativeness of the YLD estimates, particularly for conditions with limited reporting systems.

Third, when locally available data are unavailable, such as for the prevalence of certain diseases or the proportions of disease severity used in YLD estimations, this study relies on information from the GBD Study or regional estimates. Similarly, in contexts where local evidence is limited, we will adopt TMREL and RR estimates from the GBD Study for calculating PAFs. We acknowledge that incorporating more local data would enhance the accuracy of the results. To address data gaps and enhance internal consistency, DisMod II will be used to synthesize available local and international data to produce disease prevalence and incidence rates. Although this method improves comparability and coherence, the estimates remain sensitive to the assumptions and quality of the input data.

In addition, this study excluded post–COVID-19 condition from the current COVID-19 morbidity (YLD) estimates. While the analysis captures the acute disease burden, it does not account for post–COVID-19 condition health impacts experienced by individuals after recovery. Future studies should incorporate this component using updated disability weights from the GBD Study 2021 and, where available, Malaysia-specific clinical data to more comprehensively estimate the COVID-19 burden.

Finally, due to the limited variables available in this study, DALYs will only be stratified by sex and age groups. Future research could explore DALYs disaggregated by socioeconomic status and ethnicity, which would align with the United Nations’ Sustainable Development Goal (SDG) 10 of reducing inequalities.

### Implications of This Study

The findings of this study are expected to have far-reaching implications for health policy, public health planning, and research in Malaysia. The results will guide policymakers and program managers in prioritizing and strategizing health initiatives to strengthen the nation’s health care system, ultimately enhancing the health and productivity of the population [27]. By providing critical insights into the burden of disease, particularly for NCDs, the MBOD Study 2023 will inform the development of targeted interventions aimed at reducing preventable disease burdens, thereby contributing to the improvement of population health outcomes. Moreover, the data-driven approach supports the Malaysia National Strategic Plan by providing actionable data to strengthen health system resilience and guide resource allocation [28]. At the global level, this study aligns with the SDGs, particularly SDG 3, by contributing to efforts to reduce premature mortality and combat NCDs [29].

The analysis of risk-attributable burden is particularly significant, as it sheds light on the social and economic implications of disease in Malaysia. Understanding these risk factors enables more informed decision-making, prioritization of public health initiatives, and optimization of budget planning to ensure cost-effectiveness. Furthermore, this study provides evidence for targeted interventions in areas such as nutrition, physical activity, and mental health by measuring the impact of diseases and associated risk factors. It also contributes to addressing the economic burden of health care costs and reducing productivity losses. In addition, evaluating and comparing common health risk factors enables more focused, targeted public interventions aimed at the primordial and primary prevention of disease, fostering a more proactive and long-lasting approach to population health improvement [30,31].

From a methodological perspective, this study provides more accurate local estimates tailored to Malaysia’s unique context. It incorporates region-specific health priorities and underrepresented tropical diseases, ensuring that the health data are culturally relevant and applicable. Enhanced methodologies and harmonized datasets generate accurate mortality and morbidity estimates, which are essential for evaluating current health initiatives and determining priority areas for intervention. The expanded disease list, which takes into consideration Malaysia’s unique health challenges, ensures that the study remains relevant while aligning with international best practices. Finally, the study establishes a strong foundation for future research, fostering collaborations to address emerging health challenges and improve Malaysia’s overall health landscape.

### Conclusions

The MBOD Study 2023 marks a pivotal step forward in understanding Malaysia’s health challenges. By expanding the disease list and incorporating comprehensive risk factor analysis for the first time, this study delivers a nuanced perspective on mortality, morbidity, and modifiable health determinants. These advancements not only address the limitations of previous iterations but also enhance the accuracy of health data, ensuring that the data reflect Malaysia’s unique health profile, including underrepresented tropical diseases. The refined methodologies and integration of diverse datasets provide a holistic view of population health, enabling stronger evidence-based monitoring of trends, evaluation of interventions, and guidance for future research. This study establishes a vital benchmark for continuous health assessments and supports Malaysia’s commitment to improving population health outcomes and achieving sustainable health system resilience. Moving forward, it is essential to be provide annual updates on mortality data. However, the current plan is to produce the MBOD report every 4 years, in alignment with the publication of the National Health and Morbidity Survey on NCDs, to ensure the use of up-to-date prevalence data for YLD calculations.

## Abbreviations

ASEAN: Association of Southeast Asian Nations
DALY: disability-adjusted life year
DOSM: Department of Statistics Malaysia
GBD: Global Burden of Disease
MBOD: Malaysian Burden of Disease
NCD: noncommunicable disease
PAF: population attributable fraction
RR: relative risk
SDG: Sustainable Development Goal
TMREL: theoretical minimum risk exposure level
YLD: year lived with disability
YLL: year of life lost

## Data Availability

The datasets generated and analyzed during this study are not publicly available due to data protection regulations but are available from the Department of Statistics Malaysia on reasonable request and with the necessary permissions.
